# Cell-Surface Displayed Expression of Trehalose Synthase from *Pseudomonas putida* ATCC 47054 in *Pichia Pastoris* Using Pir1p as an Anchor Protein

**DOI:** 10.3389/fmicb.2017.02583

**Published:** 2017-12-21

**Authors:** Shaojie Yang, Xin Lv, Xihui Wang, Junqing Wang, Ruiming Wang, Tengfei Wang

**Affiliations:** Key Laboratory of Shandong Microbial Engineering, Qilu University of Technology (ShanDong Academy of Sciences), Jinan, China

**Keywords:** trehalose synthase, cell-surface display, Pir1p, *Pichia Pastoris*, trehalose

## Abstract

Yeast cell-surface display technologies have been widely applied in the fields of food, medicine, and feed enzyme production, including lipase, α-amylase, and endoglucanase. In this study, a *treS* gene was fused with the yeast cell-surface anchor protein gene *Pir1p* by overlap PCR, the *Pir1p-treS* fusion gene was ligated into pPICZαA and pGAPZαA and transformed into *P. pastoris* GS115 to obtain recombinant yeast strains that displays trehalose synthase(TreS) on its cell surface as an efficient and recyclable whole-cell biocatalyst. Firstly, the enhanced green fluorescence protein gene (egfp) was used as the reporter protein to fusion the *Pir1p* gene and *treS* gene to construct the recombinant plasmids containing *treS-egfg-Pir1p* fusion gene, and electrotransformed into *P. pastoris* GS115 to analyze the surface display characteristics of fusion gene by Western blot, fluorescence microscopy and flow cytometry. The analysis shown that the *treS-egfg-Pir1p* fusion protein can be successfully displayed on the surface of yeast cell, and the expression level increased with the extension of fermentation time. These results implied that the *Pir1p-treS* fusion gene can be well displayed on the cell surface. Secondly, in order to obtain surface active cells with high enzyme activity, the enzymatic properties of TreS displayed on the cell surface was analyzed, and the fermentation process of recombinant *P. patoris* GS115 containing pPICZαA-Pir1p-treS and pGAPZαA-Pir1p-treS was studied respectively. The cell surface display TreS was stable over a broad range of temperatures (10–45°C) and pH (6.0–8.5). The activity of TreS displayed on cell surface respectively reached 1,108 Ug^−1^ under *P*_AOX1_ control for 150 h, and 1,109 Ug^−1^ under *P*_GAP_ control for 75h in a 5 L fermenter, respectively. Lastly, the cell-surface displayed TreS was used to product trehalose using high maltose syrup as substrate at pH 8.0 and 15°C. The surface display TreS cells can be recycled for three times and the weight conversion rate of trehalose was more than 60%. This paper revealed that the TreS can display on the *P. pastoris* cell surface and still had a higher catalytic activity after recycled three times, which was suitable for industrial application, especially the preparation of pharmaceutical grade trehalose products.

## Introduction

Since 2000, trehalose has been accepted as a novel food ingredient under the “generally recognized as safe” designation in the United States and the European Union. It is a white, odorless powder and is a non-reducing sugar formed from two glucose units joined by an α-1,1-glycosidic bond. This special bonding can prevent Maillard reactions with proteins or amino acids (Higashiyama, 2002) and makes the compound stable at high temperatures or very acidic conditions. Because of these special properties, trehalose can be used in various industrial processes (Duong et al., 2006). Trehalose synthase (TreS) can catalyze trehalose synthesis by a one-step conversion from the low-cost substrate maltose. Therefore, this method is considered as the most economical and feasible method of production and is suitable for industrial-scale production. Hence, many researchers have attempted to increase the expression level of TreS by genetic engineering approaches, such as heterologous expression in *E. coli* (Jain and Roy, 2008; Yue et al., 2009; Kim et al., 2010; Zhu et al., 2010). Although these heterologous expression systems are successful, lack of processing mechanism after protein translation and produced endotoxin limit their application to food and medicine.

Thus far, yeast cell-surface display technologies have been widely applied in the fields of food, medicine, and feed enzyme production (Ito et al., 2004; He et al., 2008). Whole-cell biocatalysts using cell-surface displayed enzymes have great potential as industrial sorbents and sensors (Gai and Wittrup, 2007; Saleem et al., 2008; Shibasaki and Ueda, 2014). Compared with traditional immobilization of enzymes, cell-surface display not only saves tedious enzyme purification processes but can also be reused several times (Kim et al., 2002). In recent years, many cell wall proteins have been developed for surface display, such as Pir1p, Flo1p, Sed1p, Cwp2, and others (Lee et al., 2005; Wang et al., 2007; Jacobs et al., 2008; Ni et al., 2009; Kojima et al., 2012). The Pir (Protein with internal repeats) family, including cell wall proteins with internal repeats (Yang et al., 2014), can be attached to a cell wall via either an ester linkage between β-1,3-glucans and repetitive sequences located at the N-terminus, or disulfide bonds between C-terminal cysteine residues and the cell wall. The Pir1 protein belongs to the Pir group of cell wall proteins, which contain propeptides and are processed by the KeX2 protease in the Golgi apparatus (Andrés et al., 2003).

Aimed at obtaining a yeast that displays TreS on its cell surface as an efficient and recyclable whole-cell biocatalyst, the cell-surface display system of TreS in *P. pastoris* was successfully constructed using Pir1p from *Saccharomyces cerevisiae* as an anchor protein, and an enhanced green fluorescence protein (EGFP) was used to confirm the localization of TreS at the surface by Western blot analysis and flow cytometric analysis.

## Materials and methods

### Strains, plasmids, and materials

*Pseudomonas putida* ATCC47054 containing the TreS gene was obtained from the American Type Culture Collection (ATCC, Manassas, VA, USA). *P. pastoris* GS115 (Invitrogen, Carlsbad, CA, USA) was used as the host for cell-surface display. *Saccharomy cescerevisiae* CICC 32919, containing the *Pir1p* gene, was preserved in our laboratory. The pPICZαA expression vector, containing zeocin resistance, was purchased from Invitrogen (Carlsbad, CA, USA) and zeocin was obtained from Cayla(Toulouse, France). Restriction enzymes, T4 DNA ligase, and an agarose gel DNA purification kit were all purchased from TaKaRa (Dalian, China). Primers shown in Table S1 were used to amplify the target genes.

### Amplification of target genes

Genomic DNA containing the TreS gene from *Pseudomonas putida* ATCC47054 was prepared with a bacterial genomic DNA extraction kit (TaKaRa). Primers F1 and F2 were used to amplify the TreS gene (GenBank accession number 26986745). The *Pir1p* gene from the genomic DNA of *Saccharomy cerevisiae* CICC 32919 was amplified by PCR using the primers F3 and F4, which were designed based on NCBI GenBank accession number D13740. Primers F5 and F6 were used to clone the *egfp* gene from the plasmid pEGFP-N1 (Clontech, Mountain View, CA) and the*Pir1p* gene and *egfp* gene were fused with primers (F4 and F5) by overlap extension PCR. The targeted PCR products were gel-purified, inserted into pZero-BluntT vector, and transformed into *E. coli* DH5α.

### Vector construction and transformation

The PCR product containing the TreS gene from pZero-BluntT-TreS was digested with *EcoR*I and *Not*I and inserted into the *Eco*RI and *Not*I sites of pPICZαA, transformed into *E.coli* DH5α, and screened. The pZero-BluntT-EGFP-Pir1p vector containing the *egfp-Pir1p* fusion gene fragments and the pZero-BluntT-Pir1p vector containing the*Pir1p* gene fragments were digested with *Spe*I and *Xba*I, and inserted into the *Spe*I and *Xba*I sites of pPICZαA-TreS. The new recombinant plasmids named pPICZαA-TreS-EGFP-Pir1p and pPICZαA-TreS-Pir1pwere linearized using *Pme*I and then transformed into *P. pastoris* GS115 competent cells, respectively. Integration of the linearized pPICZαA-TreS-EGFP-Pir1p and pPICZαA-TreS-Pir1p plasmids into the *P. pichia* GS115 genome were confirmed by PCR using the 5′-AOX1 and 3′-AOX1 primers. Transformants were selected on YPD plates (1% yeast extract, 2% peptone, and 2% glucose) containing 100 μg zeocin ml^−1^ as the selective marker. For expression of the TreS gene under the P_GAP_ promoter, the primers G1 and G2 were used for PCR amplification of the TreS *-Pir1p* fragment from plasmid pPICZαA-TreS-Pir1p. The *P*_GAP_ promoter was cloned, using the G3 and G4 primers, from *P. pastoris* CICC 32919. The pPICZαA-TreS-Pir1p gene, without an AOX1 gene, was ligated with the *P*_GAP_ promoter by overlap PCR and the newly generated expression vector was named pGAPZαA-TreS-Pir1p.

### Localization of fusion protein by western blot analysis

The transformants were inoculated into 50 mL BMGY medium at 30°C to an optical density 600 (OD_600_) of 3 to 5. the culture were centrifuged at 4,000 × g for 5 min and resuspended in BMMY medium to an OD_600_of cells were induced for the fusion protein expression at 25°C with 100% methanol every 24 h to a final concentration of 0.5%. *P. pastoris* GS115/pPICZαA-TreS-Pir1p was used as negative control. After 96 h of induction, growth medium was collected and centrifuged at 6,000 × g for 5 min. The cells were then harvested and disrupted in buffer (20 mM Tris/HCl, pH7.5, 200 mM NaCl, and 5 mM MgCl_2_) by Bead beater (Bio-spec products, Bartlesville, USA) at 4°C. After centrifugation at 300 × g at 4°C for 5 min to remove the unlysed cells, the supernatant was collected and the cell wall fractions were isolated (Teparic et al., 2007). The remaining cell walls were incubated overnight in 30 mM NaOH at 4°C, followed by centrifugation at 10,000 × g for 5 min at 4°C. The proteins in different solutions were subjected to denaturing sodium dodecyl sulfate (SDS)-PAGE polyacrylamide gels as previously described (Tengfei et al., 2014) before transferring to nitrocellulose membrane. After 1 h, the membrane was blocked in 5% skim milk for 2 h and then probed with primary antibodies against rabbit IgG anti-EGFP antibody(1:1,000, Abcam) at 4°C overnight, followed by the secondary horseradish peroxidase conjugated goat anti-rabbit IgG antibody (1:5,000, Abcam) for 1 h. Finally, the membrane was stained in a solution containing 100 mM Tris-HCl (pH7.5), 0.8 mg/mL 3, 3′-diaminobenzidine, 0.4 mg/mL NiCl_2_ and 6 μL/mL H_2_O_2_ in the dark.

### Fluorescence microscopy observation and flow cytometry analysis

The recombinant *P. pastoris*GS115/pPICZαA-TreS-EGFP-Pir1p was grown in BMGY medium at 30°C for 16–18 h, adding methanol (a final concentration of 0.5%) to induce the expression of fusion protein. Cells were harvested at different time points (24, 48, 72, 96, 120, and 144 h) and diluted with ice-cold phosphate-buffered saline (PBS, pH7.5) to a final OD_600_ of around 0.2. Flow cytometry analysis was performed on a FACSCalibur flow cytometer (Becton Dickinson, 488 nm) using cell Questsoftware. For each detection, a total of 100,000 cells were analyzed. After induction 96 h, the recombinant *P. pastoris*GS115/pPICZαA-TreS-EGFP-Pir1p cells were collected and washed twice with phosphate buffered saline (PBS). Cells were observed using the fluorescence microscope system (NIKON Eclipse Ci-L). The *P. pastoris* GS115/pPICZαA-TreS-Pir1p induced for 96 h in BMGY was used as control.

### Fermentation of recombinant *P. pastoris* in 5 L fermenter

Fermentation studies were conducted in a 5 L fermenter (Bailun Co., Shanghai, China). *P. pastoris* GS115*/*pPICZαA-TreS-Pir1p and *P. pastoris* GS115*/*pPICZαA(GAP)-TreS-Pir1p inocula were prepared by cultivating cells for 16–18 h at 30°C in three 500 mL shake flasks containing 100 mL YPD medium. Then, inoculum was transformed to 5 L fermenters containing 2 L modified medium, which contained (per liter): KH_2_PO_4_, 42.9 g; (NH_4_)_2_SO_4_, 5 g; CaSO_4_, 0.6 g; K_2_SO_4_, 14.3 g; citric acid anhydrous, 1.92 g; MgSO_4_·7H_2_O, 11.7 g; glycerol, 40 g; and 2.0 mL PTM4 trace salt solution. Glycerol and MgSO_4_·7H_2_O were sterilized separately. The PTM4 solution contained (per liter): CuSO_4_·5H_2_O, 2.0 g; NaI, 0.08 g; MnSO_4_·H_2_O, 3.0 g; Na_2_MoO_4_·2H_2_O, 0.2 g; H_3_BO_3_, 0.02 g; CaSO_4_·2H_2_O, 0.5 g; CoCl_2_, 0.5 g; ZnCl_2_, 7 g; FeSO_4_·7H_2_O, 22 g; biotin, 0.2 g; and 1 mL concentrated H_2_SO_4_. The pH was controlled by adding 20% ammonium hydroxide and dissolved oxygen (DO) was controlled by the stirring speed (200–800 rpm) and aeration rate (0.1–2.0vvm). The fermentation process of recombinant *P. pastoris* GS115*/*pPICZαA-TreS-Pir1p comprised three phases. In the first phase (the batch phase), the culture temperature and pH were controlled at 30°C and pH 6.0 until the glycerol in the medium was used up, with a sudden increase in the DO level indicated the end of this phase. In the second phase, the glycerol-fed batch phase, the culture density was increased to an OD_600_ of approximately 150 by feeding medium containing 500 g L^−1^ glycerol and 2 ml L^−1^ PTM4 solution. Then, glycerol feeding was terminated for 30 min to exhaust the glycerol. Meanwhile, the pH was adjusted to 7.0 and the temperature was reduced to 25°C. In the third phase, the induction phase, pure methanol containing 2 ml L^−1^ PTM4 solution was fed to start the induction and the methanol concentration was adjusted to a final concentration of 0.5% once every 2 h. The DO level was maintained between 20 and 30% by adjusting the agitation and aeration rates. The fermentation process of recombinant *P. pastoris* GS115*/*pGAPZαA-TreS-Pir1p comprised the first two stages above but using 40 g L^−1^ glucose as the carbon source in the modified medium described above. Samples were taken at regular intervals and analyzed for biomass and TreS activity after fermentation. Cell density was expressed by grams of dry cell weight (DCW) per liter, which was obtained by centrifuging 10 mL samples in centrifuge tubes at 8,000 × g for 10 min, washing twice with deionized water, and drying the pellets to a constant weight at 105°C for about 150 min. The dry weight of the pellets was measured every half hour.

### Effect of temperature and pH on surface-displayed TreS activity and stability

The harvested yeast cells obtained through enzymolysis by laminarinase (name Pure Tres expressed by *P. pastoris* GS115 and cell surface display Tres expressed by pGAPZαA-TreS-Pir1p) were mixed with 10 mL pure maltose solution (600 g L^−1^) and then diluted to 20 mL with fresh phosphate buffer. The maltose final concentration in the reaction system was adjusted to 300 g/L. The reaction mixture was incubated at different temperatures (10°C, 15°C, 20°C, 25°C, 30°C, 35°C, 40°C, 45°C, 50°C, 55°C, 60°C, 65°C, 70°C, 80°C) and pH (pH 3, pH 4, pH 5, pH5.5, pH 6, pH6.5, pH7, pH7.5, pH8, pH8.5, pH9, pH10) for 1 h and then heated to 100°C for 10 min. Then, the weight content (%, w/v) of trehalose in the samples were identified by comparing with the trehalose standard curves of various concentrations determined by high-pressure liquid chromatography (HPLC, Shimadzu-GL Sciences, Japan). The apparatus was equipped with an Inertsil-NH_2_ column (4.6 × 250 mm, Shimadzu) at 40°C. Separation was achieved by pumping acetonitrile: water (75:25 v:v) at a flow rate of 1.0 mL min^−1^ for 20 min. One unit (U) of TreS was defined as the amount of enzyme required to produce 1 μmol trehalose per hour. Optimum temperature and pH were determined by individually changing the conditions of the surface-displayed TreS activity assay (temperature 10–80°C and pH 3.0–10.0).

## Results

### Construction of cell-surface display vectors

The plasmids pPICZαA-TreS-EGFP-Pir1p, pPICZαA-TreS-Pir1p and pPICZαA(GAP)-TreS-Pir1p were constructed as described in the Methods. The construction process for the recombinant vectors is shown in Figure S1. PCR analysis was performed to determine whether the *TreS* and *pir1p* fusion genes were integrated into the *P. pastoris* genome. The resulting PCR product lengths revealed that the TreS and *pir1p* fusion gene was correctly inserted into the *P. pastoris* genome (Figure S2).

### Confirmation of fusion protein on cell-surface display

**Figure 1:**
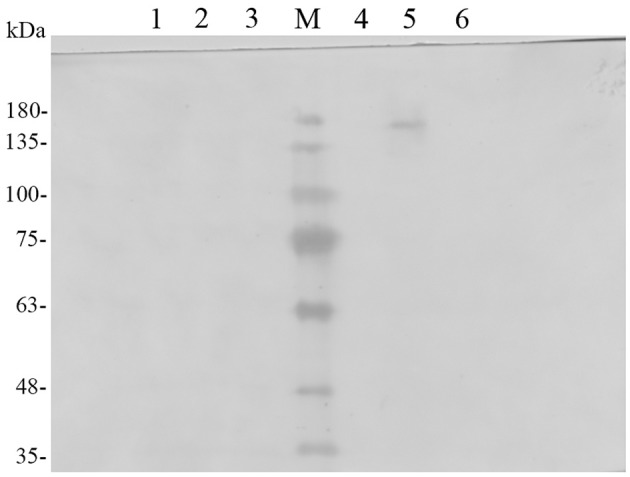
Western blot analysis of the localization of the TreS-EGFP-Pir1p fusion protein. Lane M: protein molecular marker, Lane 1: *P. pastoris* GS115/Pir1p-TreS concentrated growth media, Lane2: supernatant of *P. pastoris* GS115/Pir1p-TreS cell wall removed, Lane3: Proteins released from the cell walls of *P. pastoris* GS115/Pir1p-TreS, Lane 4: supernatant of *P. pastoris* GS115/Pir1p-EGFP-TreS cell wall removed, Lane5: Proteins released from the cell walls of *P. pastoris* GS115/Pir1p-EGFP-TreS. Lane 6: *P. pastoris* GS115/Pir1p-EGFP-TreS concentrated growth media.

To confirm that Pir1p-EGFP-TreS fusion protein was successfully expressed on recombinant *P. pastoris* GS115 cell surface. The recombinant *P. pastoris* GS115/pPICZαA-TreS-EGFP-Pir1p and *P. pastoris* GS115 as control were induced in BMMY medium for 96 h, the cells were collected to extract the cell wall proteins. The extracts from the purified cell walls, and concentrated samples of cell contents and growth medium from recombinant *P. pastoris* GS115/pPICZαA-TreS-EGFP-Pir1p and *P. pastoris* GS115 were analyzed by Western blot using an EGFP antibody. The fusion proteins in the extracts from the cell of *P. pastoris*GS115/pPICZαA-TreS-EGFP-Pir1p and *P. pastoris* GS115 were detected in Figure 1 (Lanes 2 and 5), indicating that they may attach to the cell wall as previously reported (Wang et al., 2008). No specific polypeptides could be detected from the concentrated growth and the supernatant of cell wall removed from *P. pastoris* GS115/pPICZαA-TreS-EGFP-Pir1p and *P. pastoris* GS115 (Figure 1, Lanes 1, 3, 4, 6), indicating that the fusion was displayed mainly on the cell wall. The TreS-EGFP-Pir1p fusion protein showed a band of approximately 175–180 kDa, which was larger than the predicted 130-kDa size, which may be attributed to glycoslated as reported.

**Figure 2:**
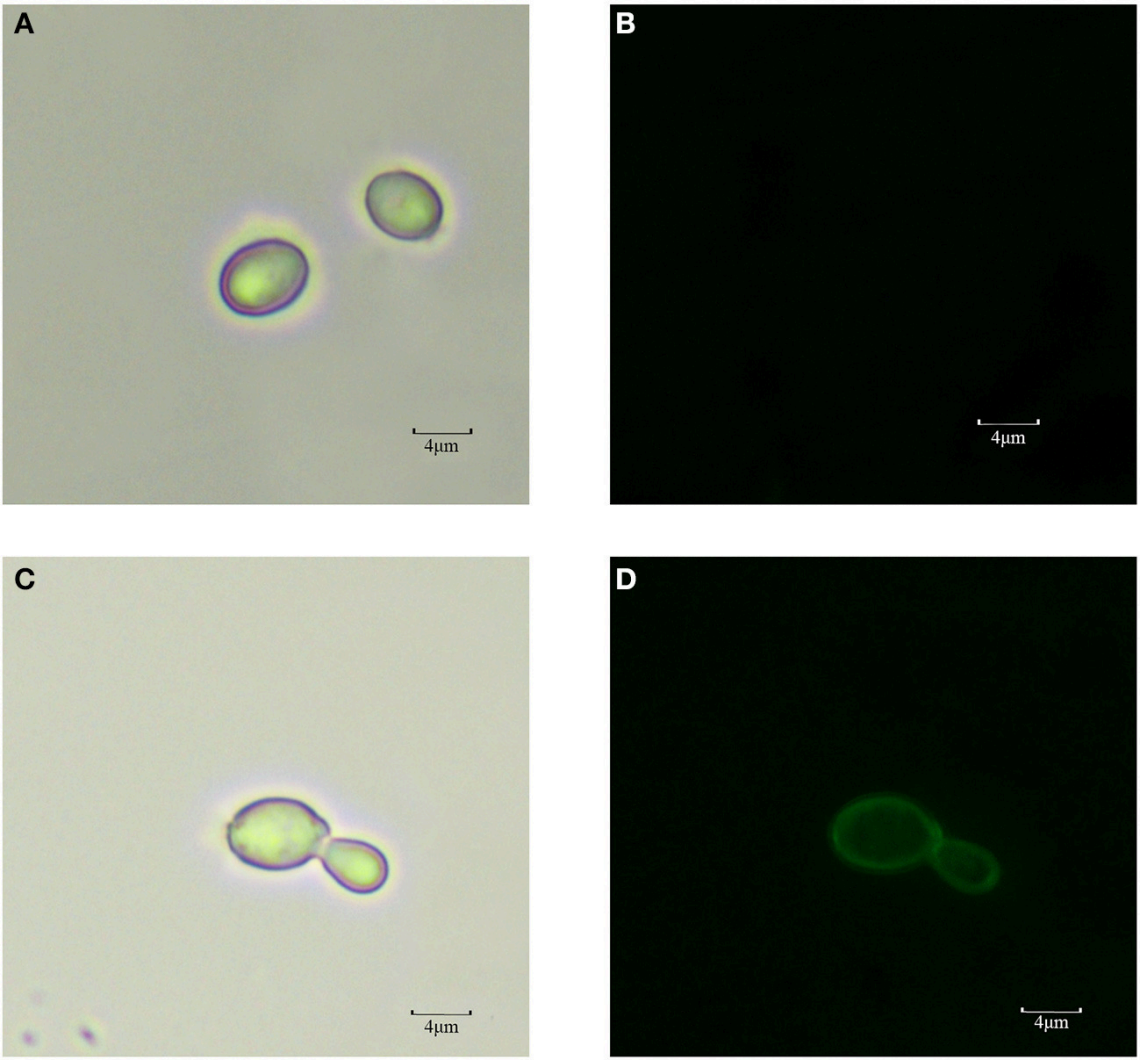
Observation of *P. pastoris* GS115 and *P. pastoris* GS115/Pir1p-EGFP-TreS induced for 48 h in medium by fluorescent microscopy. **(A)** *P. pastoris* GS115 under normal white light. **(B)** *P. pastoris* GS115 under the green emission filter. **(C)** *P. pastoris* GS115/pPICZαA*-*TreS-EGFP-Pir1p under normal white light. **(D)** *P. pastoris* GS115/ pPICZαA*-*TreS-EGFP-Pir1p under the green emission filter.

**Figure 3:**
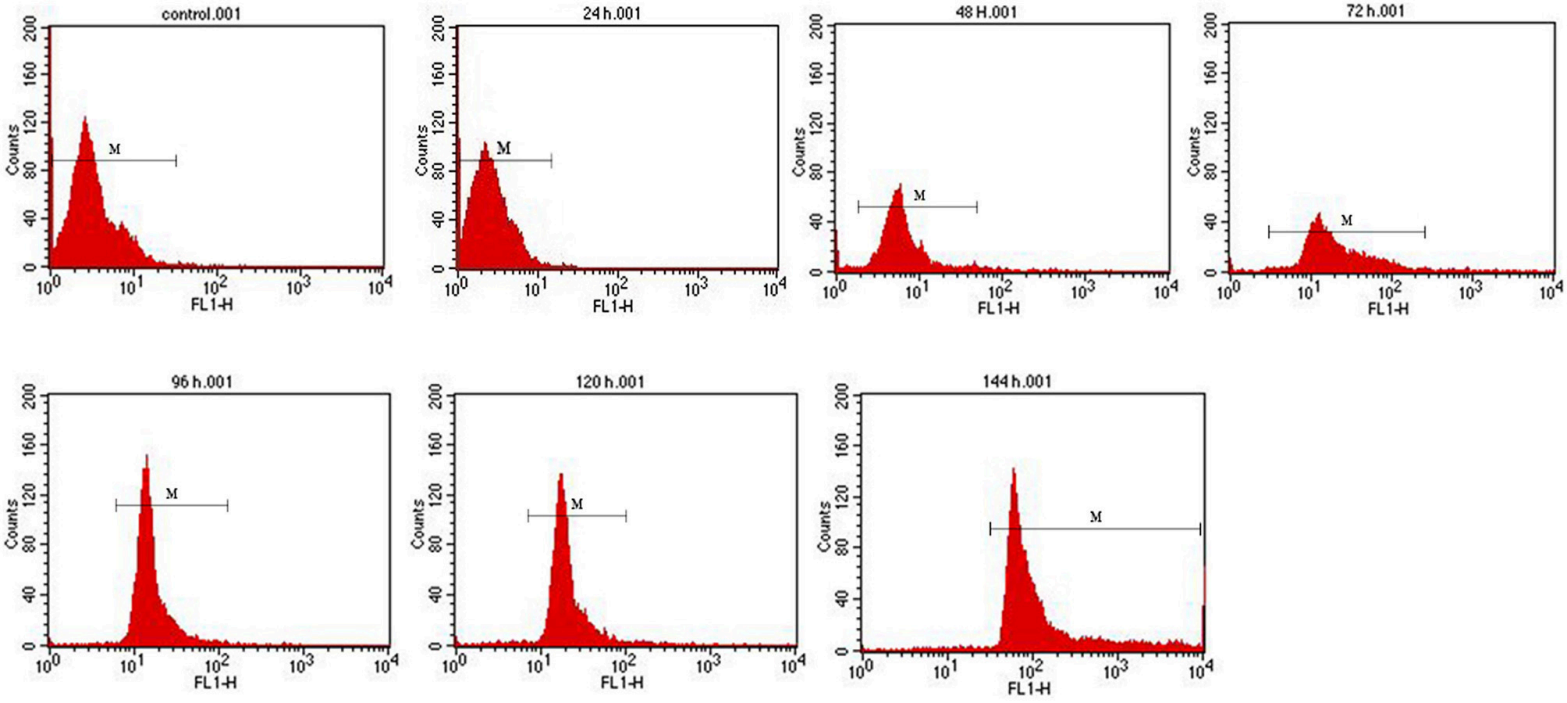
Flow cytometry analysis of recombinant *P. pastoris* GS115/Pir1p-EGFP-TreS surface display cells at different induction times. Recombinant *P. pastoris* GS115/Pir1p-TreS was used as control.

The cells of recombinant *P. pastoris* GS115/pPICZαA*-*TreS-EGFP-Pir1p and *P. pastoris* GS115 (blank control) were observed by fluorescence microscopic, respectively. Green fluorescence was clearly observed on the cell surface *P. pastoris* GS115/pPICZαA*-*TreS-EGFP-Pir1p and no green fluorescence was observed on the *P. pastoris* GS115. This result showed that the fusion protein TreS-EGFP was successfully anchored and displayed on the cell surface of *P. pastoris* GS115 (Figure 2). The presence of TreS-EGFP on the surface was further verified by flow cytometric analysis. Only the cells expressing TreS-EGFP from *P. pastoris* GS115/pPICZαA*-*TreS-EGFP-Pir1p could be detected and the control *P. pastoris* GS115 could not be detected. This analysis showed that the intensity of the fluorescence gradually increased after 24 h with the extension of induction time (Figure 3), suggesting the successful expression of the fusion protein on the cell surface of *P. pastoris* GS115. All together, the results indicated that the expressed TreS-EGFP fusion protein was anchored at the cell wall, suggesting that the Pir1p anchoring motif can be successfully used as anchoring protein to display Pir1p-TreS on the *P. pastoris* GS115 surface.

### Properties of the displaying TreS in *P. pastoris*

**Figure 4:**
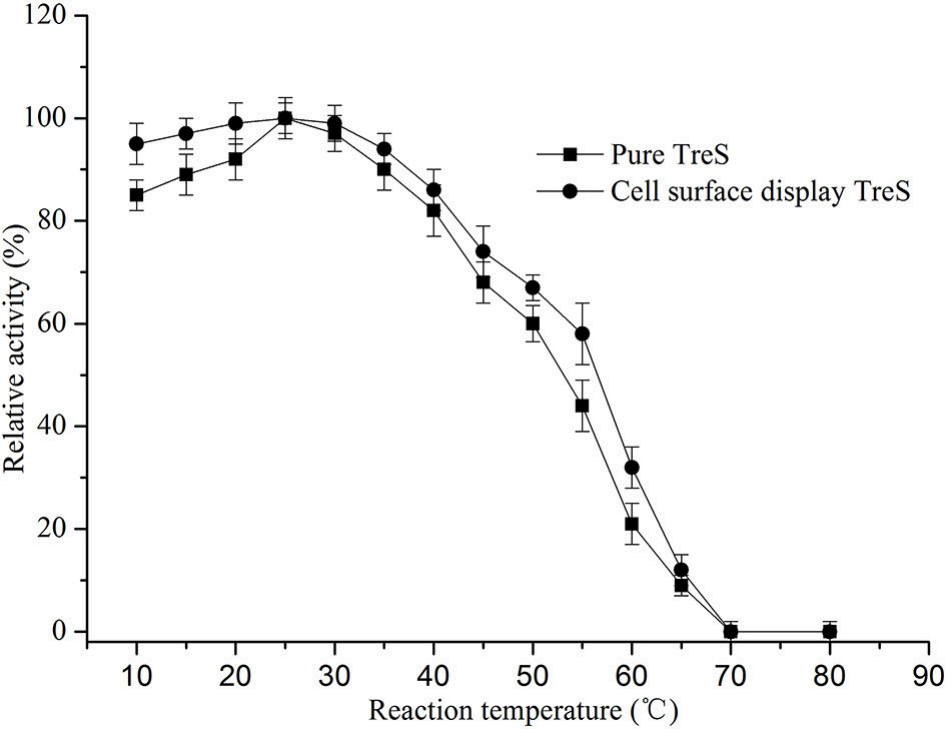
Effects of temperature on the activity of purified TreS and cell-surface displayed TreS at pH8.0. Each value and error bar represents the mean of three independent experiments and its standard deviation.

**Figure 5:**
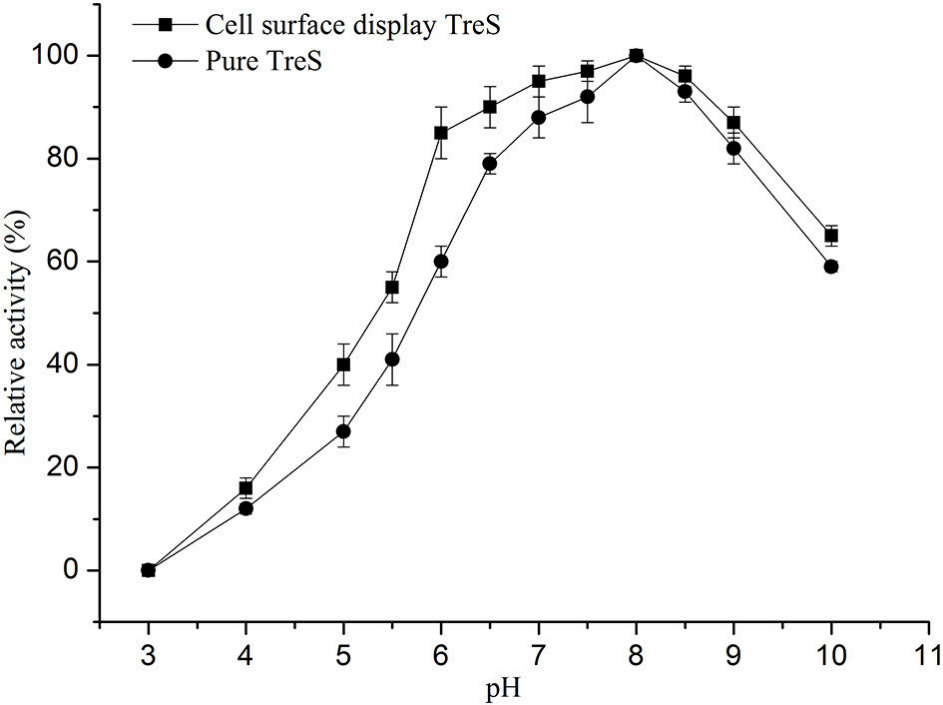
Effects of pH on the activity of purified TreS and cell-surface displayed TreS at temperature 25°C. Each value and error bar represents the mean of three independent experiments and its standard deviation.

In order to keep the enzyme activity better during the fermentation and the transformation process. The characterization of surface display TreS in relation to temperature and pH was investigated in this study, as shown in Figures 4, 5. The *P. pastoris* GS115 displaying TreS exhibited over a broad range of pH (6.0–9.0) and temperature (10–50°C) with the optimum pH 8.0 and temperature at 25°C. When the temperature is 25°C and pH is 8.0, the enzyme activity of TreS was defined as maximum activity to produce 1 μmol trehalose per hour using pure maltose (300 g/L) as substrate. The displayed TreS kept more than 95% of its maximum activity at 15°C and pH7.0, but the relative activity of displayed TreS was lower than 40% when the temperature went over 60°C or the pH was less than 6.0.

### Fermentation of recombinant *P. pastoris*

**Figure 6:**
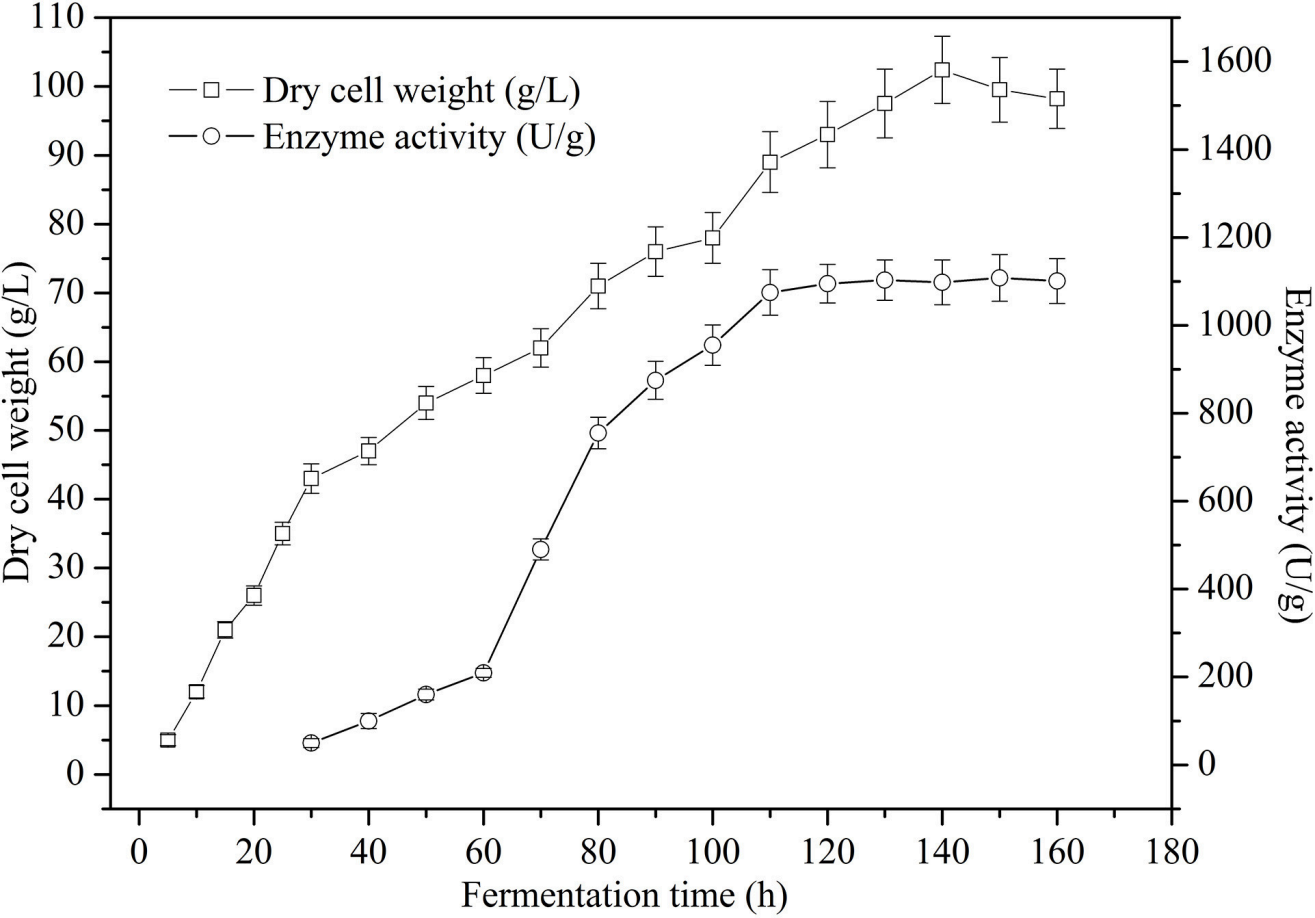
Time course of dry cell weight and cell-surface displayed TreS activity from recombinant *P. pastoris* GS115/pPICZαA -TreS-Pir1p grown in a 5 L fermenter. Each value and error bar represents the mean of three independent experiments and its standard deviation.

To obtain a high cell biomass and TreS cell-surface activity, fed-batch studies were conducted in 5 L fermenters using recombinant *P. pastoris* GS115*/*pPICZαA-TreS-Pir1p. TreS expression under the *P*_AOX1_ promoter was controlled using methanol as an inducer. Following an increase in DO, due to a carbon source limitation after approximately 20 h of fermentation, the glycerol fed-batch process was conducted for another 4 h until the DCW biomass reached about 35 g L^−1^. After glycerol depletion, the culture respiratory activity decreased with a fast DO spike. The methanol was feed every 24 h. After methanol-initiated induction, the culture respiratory activity recovered and the DO concentration decreased. During the first 4 h of the induction phase, methanol accumulated in the fermenter and DO levels were erratic while the culture adapted to the methanol. After methanol adaptation, a constant feed rate was maintained throughout the remainder of the fermentation. We also found that the methanol concentration should be kept constant to maintain limited growth after adaptation and then increased gently 60 h after induction, signaling that fermentation had stopped. At the process of the fermentation, the culture DCW reached 102 g L^−1^at 130 h and the enzyme activity reached 1,108 U g^−1^at 150 h (Figure 6). Though the DCW increased during the induction phase, TreS expression accumulated progressively during the first 60 h after methanol induction and subsequently declined.

**Figure 7:**
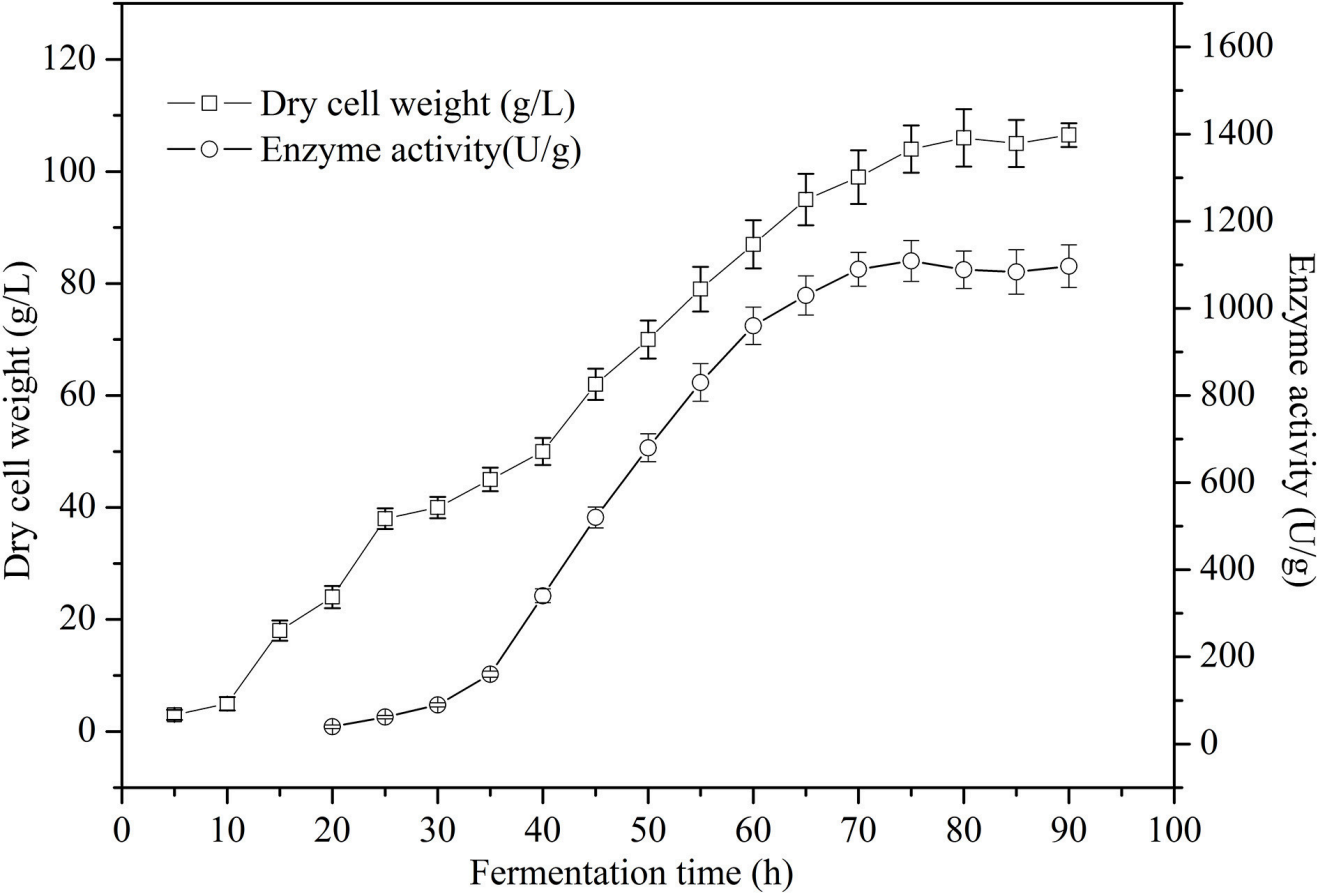
Time course of dry cell weight and cell-surface displayed TreS activity of recombinant *P. pastoris* GS115/pZαA(GAP)-TreS-Pir1p grown in a 5 L fermenter using glucose as carbon source. Each value and error bar represents the mean of three independent experiments and its standard deviation.

Product formation appeared to be greater in the *P*_GAP_ promoter constitutive expression system. Therefore, a higher cell density would help to achieve an even higher product concentration. Fed-batch studies were carried out in 5 L fermenter in order to obtain a high cell biomass. As shown in Figure 7, the maximum TreS activity and DCW reached 1,109 U g^−1^ at 75 h and 104 g L^−1^ at 80 h respectively, using glucose as a carbon source. The cell viability remained nearly 99% after 80 h of culture in the 5 L fermenter.

### Production of trehalose by surface-displayed TreS

To reduce the cost of syrup and improve the use efficiency of surface display TreS. The high maltose syrup containing 90.5 ± 1.2% maltose, 3.3 ± 0.8% glucose, 1.3 ± 0.2% maltotriose, 4.5 ± 0.5% other polysaccharides (Shandong Lvjian Biological Technology Co. Ltd.) was used as substrate at a final maltose concentration of 300 g L^−1^, and the temperature at 15°C and pH 8.0 was used in the conversion process for reducing the bacterial contamination and autolysis of yeast cells. The sterilized high maltose syrup was mixed with TreS displayed on the cell-surface of *P. pastoris* GS115 (200 ± 10.0 U g^−1^ maltose) at 15°C and 0.01 M sterilized phosphate buffer (pH 8.0) in a 10 L bioconversion and stir at 60 rpm for conversion(Figure S3). The first time, trehalose content (w/v) in conversion system reached equilibrium at 16 h, accounting for about 64% (v/v) of total maltose in conversion solution, and the enzyme activity retained more than 93% of its initial enzyme activity of per gram yeast cell. For further utilization of enzyme activity, after the first conversion, the yeast cells of surface display TreS were centrifuged at 3,000 × g for 5 min, collected under aseptic condition, added to the conversion tank again, and mixed with the same amount of sterilized high maltose syrup for the second conversion until the trehalose content in conversion solution reached equilibrium. After the second conversion, the weight conversion rate of trehalose reached about 63.2% of total maltose in conversion solution at about 20 h and the enzyme activity retained more than 85% of its initial activity. After the third conversion, the weight conversion rate of trehalsoe reached about 63.15% of total maltose in conversion solution at about 25 h and the enzyme activity retained more than 68% of its initial activity. Meanwhile, autolysis of yeast cells increased gradually when the fourth conversion, affecting the stability of transformation system and increasing the probability of bacterial contamination and the process cost of syrup in the late stage, which would influence the quality of products. Therefore, the cell-surface displayed TreS was recycled three times.

## Discussion

Pir1p is reported to improve the performance of the catalyst by displaying an enzyme anchored by the C-terminus without hindering the catalytic site, which has been applied to display the reporter protein GFP and human α-1-antitrypsin (Khasa et al., 2011). As determined by Western blot and fluorescence microscopy, the fusion protein Pri1p-EGFP-TreS was successfully expressed throughout the yeast cell surface. No specific polypeptides could be detected from the concentrated growth medium and the supernatant of cell wall removed, indicating that the fusion proteins were retained mainly at the cell wall. The molecular mass of the Pir1p-EGFP-TreS was larger than the predicted values, implying that the fusion protein may be glycosylated. This result is consistent with a previous report (Wang et al., 2008). The fluorescence intensity of the fusion proteins gradually increased after 24 h by flow cytometry analysis, suggesting the amount of fusion protein displayed on the cell surface will be increased with the induction time, and eventually reached a more stable display quantity. The increase of enzyme activity after fermentation by recombinant *P. pastoris* GS115/Pir1p-TreS also proved this point.

Heterologous expression did not change the properties of the enzyme; however, the characteristics of the biocatalyst can be affected by immobilization (Moura et al., 2015). A study by Wang et al. (2014) revealed that the *treS* gene expressed in *E. coli* BL21 (DE3) had stable activity at a temperature of 55°C, a pH of 7.4, and catalyze 59% maltose into trehalose with a substrate concentration of 30%. The higher temperature can effectively avoid contamination of miscellaneous bacteria, but it also caused certain enzyme activity loss. On the other hand, the maximum trehalose yield (approximately 81%) catalyzed by the TreS from *Thermus thermophilus* HB-8 (ATCC 27634), expressed in *E. coli* BL21 (DE3), was obtained at an optimum temperature of 65°C and a pH of 7.0 and was independent of substrate concentration (Wei et al., 2013). In addition, the trehalose conversion rate of 75% at 30°C by TreS (9,234 U/mL), expressed in *E. coli* BL21 (DE3), in a 50 L fermentor was reached with 6% glucose generated as byproduct using maltose syrup (30 wt%) as substrate in the bioreactor system via whole-cell biocatalysis (Song et al., 2016). However, the loss of plasmid often occurs and the constructed strain is extremely unstable in prokaryotic expression system (Le and Dobson, 1997; Rosa et al., 2005). In industrial production, the production of trehalose synthase is limited due to the existence of fragmentation and endotoxin (Tengfei et al., 2014). In our study, *P. pastoris* GS115 displaying TreS exhibited optimal activity at a temperature of 25°C, pH 8.0, with a substrate concentration of 300 g/L (maltose determined by HPLC). When the temperature was over 60°C or the pH was less than 6.0, the relative activity of displayed TreS activity was lower than 40%. Our results were inconsistent with the previous study (Wei et al., 2013; Wang et al., 2014), which may be attributed to the immobilization. Meanwhile, the conversion rate of trehalose reached about 63.2% of total maltose at about 20 h and the enzyme activity retained more than 85% of its initial activity. The cell-surface displayed TreS can be recycled three times. This results are better than previous studies (Wei et al., 2013; Tengfei et al., 2014; Wang et al., 2014).

*P*_AOX1_ is a strongly regulated promoter from alcohol oxidase I and has been chosen for production of a variety of recombinant proteins in *P. pastoris*. *P*_GAP_, a strong constitutive promoter, has also been used for constitutive expression of some heterologous proteins (Zhang et al., 2009). Several studies have suggested that *P*_GAP_ is more efficient than *P*_AOX1_ for the expression of eukaryotic heterologous proteins (Döring et al., 1998; Delroisse et al., 2005). Nevertheless, some studies revealed that using *P*_AOX1_ as promoter resulted in higher production than using the *P*_GAP_ promoter (Boer et al., 2000; Vassileva et al., 2001). In the *P*_AOX1_ expression system, methanol is applied to induce protein expression. However, methanol is a toxic substance that may not be appropriate for the production of food products and easily causes a fire hazard, especially in large-scale fermentations (Wang et al., 2012). Additionally, the time required to achieve peak product concentration lasts longer (Qiao et al., 2010) and the fermentation process needs to shift the carbon source from glycerol to methanol (Zhang et al., 2009). In the *P*_GAP_ expression system, cell growth and heterologous protein production occur simultaneously in the process of fed-batch fermentation. Also, the hazard and cost associated with the storage and delivery of a large volume of methanol are eliminated. In this study, we successfully expressed the *treS* gene from *Pseudomonas putida* ATCC47054 under the control of *P*_AOX1_ and *P*_GAP_ on the cell surface of *P. pastoris*. The highest activity of recombinant TreS under the control of *P*_AOX1_ reached above 1,100 U g^−1^ in a 5 L fermenter after 140 h of culture, and the recombinant TreS produced in the *P*_GAP_ promoter expression system exhibited a maximum activity of above 1,109U g^−1^ using glucose as the carbon source in a 5 L fermenter after 75 h of culture. Our results further demonstrated that the *P*_GAP_ expression system can be an alternative to the *P*_AOX1_ system for heterologous expression of TreS in *P. pastoris*, without obvious differences in expression level.

## Conclusion

We successfully constructed the recombinant *P. pastoris* GS115/Pir1p-EGFP-TreS to prove the fusion protein displayed on the *P. pastoris* GS115 cell surface using Pir1p as anchor protein by Western blot localizaton, fluorescence microscopy observation and flow cytometry analysis. The recombinant *P. pastoris* GS115/Pir1p-TreS can successfully display TreS and used to produce trehalose. The expression levels of TreS obtained in the 5 L fermenters indicate that the *P*_GAP_ system is a suitable alternative to the *P*_AOX1_ system for large-scale production of recombinant TreS in *P. pastoris* GS115 by a high cell density culture, and the enzyme activity reached above 1,100 U g^−1^. It can be concluded that constitutive TreS expression in a continuous culture of *P. pastoris* may be easy to scale up and has good prospects for decreasing production costs for large-scale industrial fermentations.

## Author contributions

TW and RW designed the project. SY, XL, and XW performed experiments. SY, XL, XW, and JW discussed and interpreted results. SY, XL, and TW wrote the manuscript. All authors agreed to be accountable for accuracy, integrity and appropriateness of the manuscript. Authors initials in order: SY, XL, XW, JW, RW, TW.

### Conflict of interest statement

The authors declare that the research was conducted in the absence of any commercial or financial relationships that could be construed as a potential conflict of interest.

## Supplementary material

Figure S1 The construction process of the recombinant vectors.

Figure S2 Results of the pPICZαA-TreS-Pir1p recombinant plasmid restriction enzyme analysis. M: Trans5K DNA Marker; 1: Triple digestion of recombinant plasmid pPICZαA-TreS-Pir1p.

Figure S3 Schematic diagram of trehalose system prepared by surface display cells.

Table S1 The primers used in this study.
