# Supplementary figures and images for: Cell-Surface Displayed Expression of Trehalose Synthase from Pseudomonas putida ATCC 47054 in Pichia Pastoris Using Pir1p as an Anchor Protein
Source: Front Microbiol. 2017 Dec 21;8:2583. doi: 10.3389/fmicb.2017.02583 (PMC5742630; doi:10.3389/fmicb.2017.02583)

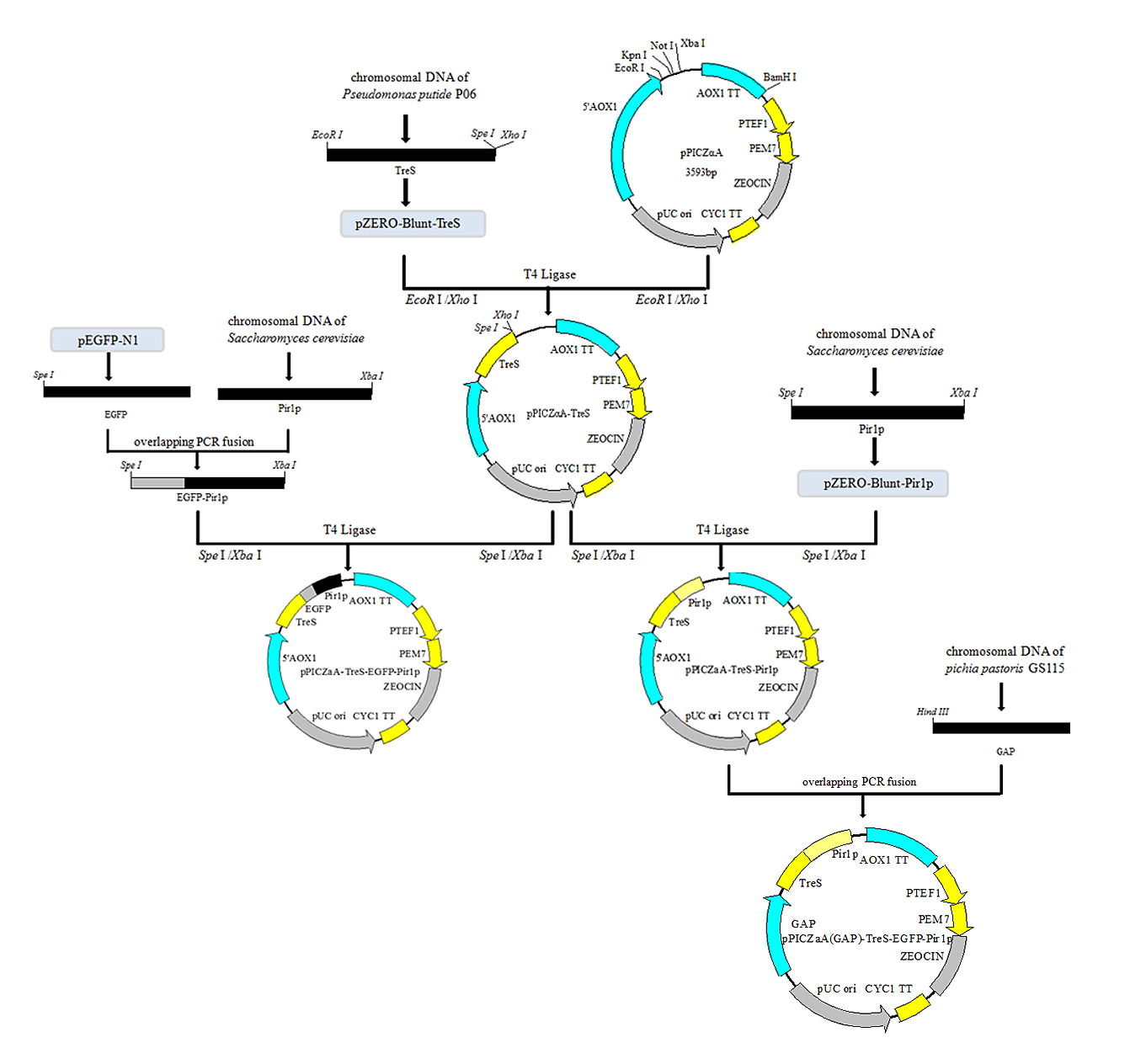

Supplement: Figure S1 — The construction process of the recombinant vectors. [file Image1.JPEG]

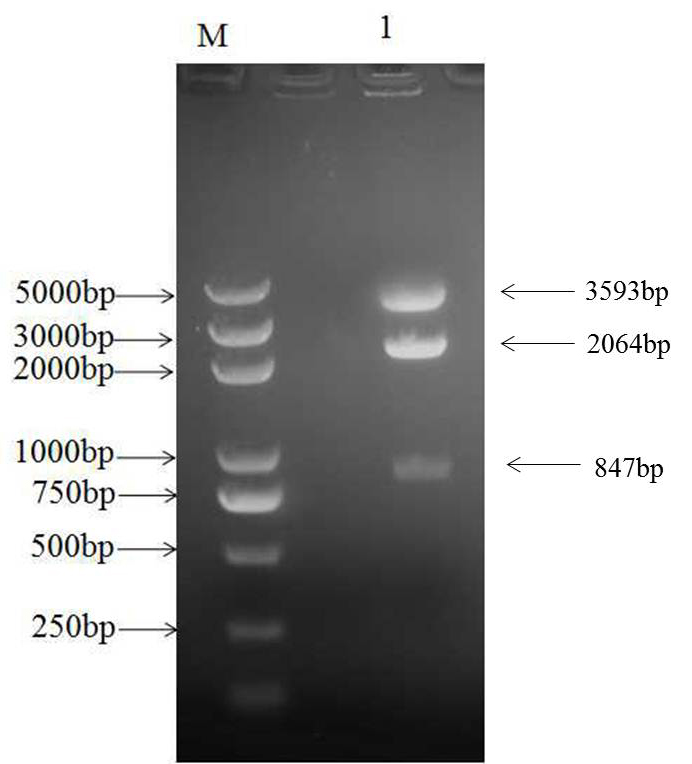

Supplement: Figure S2 — Results of the pPICZαA-TreS-Pir1p recombinant plasmid restriction enzyme analysis. M: Trans5K DNA Marker; 1: Triple digestion of recombinant plasmid pPICZαA-TreS-Pir1p. [file Image2.JPEG]

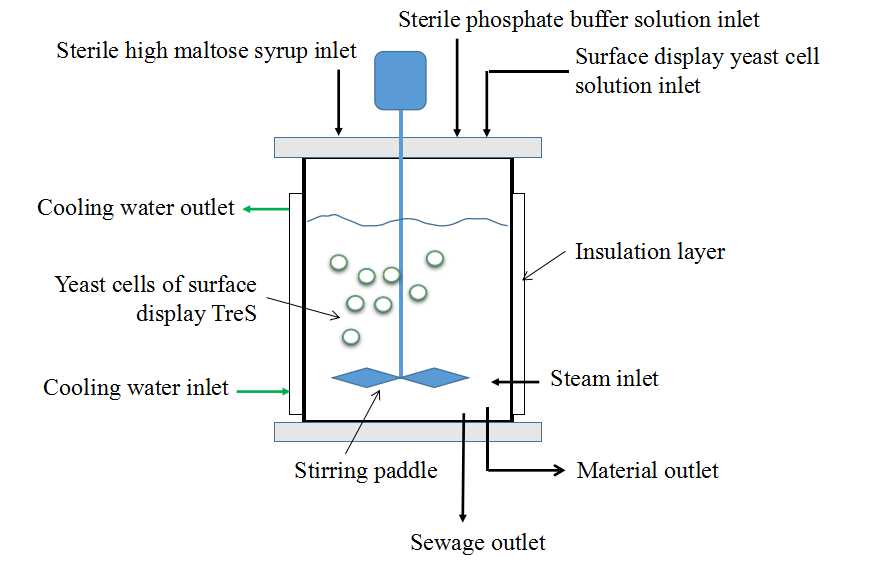

Supplement: Figure S3 — Schematic diagram of trehalose system prepared by surface display cells. [file Image3.JPEG]
