# Supplementary material for: Cell-Surface Displayed Expression of Trehalose Synthase from Pseudomonas putida ATCC 47054 in Pichia Pastoris Using Pir1p as an Anchor Protein
Source: Front Microbiol. 2017 Dec 21;8:2583. doi: 10.3389/fmicb.2017.02583 (PMC5742630; doi:10.3389/fmicb.2017.02583)
Supplement: Table S1 — The primers used in this study. [file Table1.DOCX]

| Primer | Gene sequence |
| --- | --- |
| F1 | 5`- CCG*GAATTC*ATGACCCAGCCCGACCCGTC -3`(*EcoR* I *italic*) |
| F2 | 5`- GC*TCTAGA*ACTAGTAACATGCCCGCTGCTGTTGAC -3`(XbaI *italic*, *Spe*I *wave line*) |
| F3 | 5`- TCTTCCTCCTCCTCTTCCTCCTCCGCCGCTGCTATCTCTCA -3` |
| F4 | 5`- GC*TCTAGA*TTAACAGTTGAGCAAATCGATAGC -3`(*Xba*I *italic*) |
| F5 | 5`-GG*ACTAGT*GGAGGCGGGAGAGGAGGCGGAAGAATGGTGAGCAAGGGCG-3`(*Spe*I *wave line*) |
| R1 | 5’-GG*ACTAGT*CAGAGAGCCGCTGCTAT-3’(*SpeI* *italic*) |
| R2 | 5’-GC*TCTAGA*TTAACAGTTGAGCAAATCGAT (*XbaI italic*) |
| G1 | 5`- CCCC*AAGCTT*AAACGATGAGATTTCCTTCAA -3`(HindIII *italic*) |
| G2 | 5`- ACAAAAAAGGATCGATCTCATGACCAAAATCCC -3` |
| G3 | 5`- ATTTTGGTCATGAGATCGATCCTTTTTTGTAGAAATGTC -3` |
| G4 | 5`- GGGG*AAGCTT*TGTGTTTTGATAGTTGTTCA -3`(HindIII *italic*) |

Table1 The primers used in this paper
